# A Picture is Worth 1000 Words: Using Graphic Novels to Support Health Professions Education

**DOI:** 10.1007/s40670-026-02871-9

**Published:** 2026-10-01

**Authors:** Karen (Beth) Marcellas, Melissa Myers, Dina Kurzweil

**Affiliations:** 1 Education & Technology Innovation (ETI) Support Office, Uniformed Services University of the Health Sciences (USU), Bethesda, MD USA; 2 Military & Emergency Medicine Department, Uniformed Services University of the Health Sciences (USU), Bethesda, MD USA; 3 Education & Technology Innovation (ETI) Support Office, Military & Emergency Medicine Department, Uniformed Services University of the Health Sciences (USU), Bethesda, MD USA

**Keywords:** Health Professions Education, Faculty Development, Evidence-based instruction, Graphic medicine, Assessment

## Abstract

The article illuminates the communicative power of graphic novels and the instructional rationale for their use for health professions education (HPE), particularly for enhancing critical thinking, empathy, and interpersonal skills. The article also provides a methodological framework for creating graphic novels that incorporates Generative Artificial Intelligence (GenAI) for narrative codification and visual synthesis. It outlines a two-phase process: Phase I (Architecture), focused on conceptual design, and Phase II (Construction), which utilizes a "Human-AI-Human" (H-AI-H) workflow to streamline production while maintaining pedagogical integrity.

**Supplementary Information:**

The online version contains supplementary material available at https://doi.org/10.1007/s40670-026-02871-9.

## Introduction

While comic books and cartoons are frequently seen as “light reading,” there is a parallel tradition of more serious works that combine words and graphics to tell a more sustained, detailed, and complex story—works such as Marjane Satrapi’s *Persepolis* [1] and Art Spiegelman’s *Maus* [2]. Will Eisner used the term graphic novel to describe such works, which combine sequential art with written text in a way that can convey a deeper meaning than either could do separately [3, 4]. This combination of two types of storytelling—visual and textual—can be used effectively in a wide variety of educational contexts [5, 6]. There is a history of using comics and graphic novels in both formal and informal military education [3, 7], though in this context they have frequently been used more for didactic purposes than to support critical thinking and the development of interpersonal and communication skills.

This article makes a case for the suitability and practicability of using graphic novels for health professions education (HPE). It illuminates the communicative power of graphic novels, describes the instructional rationale for using graphic novels in teaching and learning, and explains some of their strengths in the context of HPE. Additional exploration of their benefits comes from a discussion of the use of graphic novels to prepare faculty to serve as instructors during a live simulation. Finally, the article provides guidance about how to create graphic novels for instructional use, laying out the steps in the development process and identifying ways that generative artificial intelligence (GenAI) can be used to support and enhance the process.

## The Communicative Power of Graphic Novels

The communicative power of graphic novels is illuminated by Clark and Mayer’s multimedia principle [8, 9], media theorist Marshall McLuhan’s explanations of “hot” and “cool” media [4], and John Keller’s Attention, Relevance, Confidence, and Satisfaction (ARCS) theory of motivation [10]. Keller’s model posits that learners are motivated by instructional material that gains their attention, makes clear its relevance, gives them confidence that they can master the objectives, and provides satisfaction by reinforcing the learners’ knowledge and their success in attaining the objectives.

Clark and Mayer’s multimedia principles are grounded in Mayer’s cognitive theory of multimedia learning (CTML), which posits that the brain processes or “codes” images and text separately [11]. According to CTML, educational materials designed to take advantage of both processing systems can enhance learning, while those that present too much information to one or the other processing systems, thus increasing cognitive load, or the amount of processing required, can hinder learning [11]. Mayer’s principles also build on dual coding theory’s assertion that, although visual and verbal information processing have separate pathways in the brain, there are interconnections between them [12]. Pairing pictures and words in instructional materials promotes learning and retention, as learners form connection between the two representations [9, 12–15].

While Mayer examined the way that instructional graphics are processed in the brain, communication theorists considered the way that people interact with and consume such media. Short and Reeves [4] cite Marshall McLuhan’s (1964) explanations of “hot” media, such as photographs, which are dense with information, and “cool” media, such as drawings, which contain less dense information, to explain the way readers experience what they read and see. Because they have space limitations, graphic novels and comics tend to use minimalistic graphics and limited text. As a result, readers must “fill in the blanks” and participate actively in interpreting their meaning [4, 16–18]. This process can be particularly relevant in education in the healthcare professions: “As they interpret the incomplete visual data inherent to comics and transform this into a coherent story, they mimic the diagnostic process in medicine—where the physician is supplied with amorphous information, and must make inferences and draw conclusions to produce an accurate diagnosis” ([19], p. 474). This active reading process can also enhance recall, which is in keeping with Mayer’s findings [20].

The ability of graphic novels and comics to convey layers of meaning via visuals, text, and the interplay between the two, makes them good vehicles for telling complex stories [5, 21]. Such complex stories can help motivate learners, gaining their attention and helping them see the relevance of the material [22–26], in keeping with Keller’s ARCS model of motivation [10]. Studies have found that learners of many ages find them to be compelling and may even prefer them to other “traditional” types of learning materials [23–26].

## Educational Uses of Graphic Novels

Because of the communicative power of graphic novels, they can support HPE in a number of ways.

One of the strengths of graphic novels is their ability to ground learning in a specific context. Realistic graphics are a hallmark of graphic novels, and they can help learners familiarize themselves with an unfamiliar environment [6]. The educational value sometimes lies as much in what is left out as in what is shown. Graphic novels can help learners by presenting a simplified version of a complex scene, eliminating some unnecessary detail in order to keep the focus on the most relevant elements of the scene [16, 27].

Well-built instructional materials can provide scaffolding, the temporary, adjustable supports provided to help a learner perform a task within their “zone of proximal development”—a range of tasks they cannot yet manage independently but can achieve with guidance [28]. Scaffolding is critical because it reduces extraneous cognitive load—the unnecessary mental effort spent on irrelevant environmental details. This cognitive load management allows learners to focus their limited working memory on relevant assessment markers and germane learning processes [29, 30]. This is particularly vital in specialized fields where the design of instruction needs technical accuracy and realism, such as anatomy or surgery [16, 27].

In some cases, researchers have found that elements of graphic novels or comics are superior to text, because they are more efficient in their presentation of information [31, 32]. The ability to incorporate custom-designed visuals means that graphic novels and comics can help attain pedagogical goals efficiently for complex knowledge areas when the design of instruction needs much technical accuracy and realism in essential components (for different modalities), such as medical education, health psychology, and military procedures and leadership [7, 33–35]. Graphic novels can also be helpful in health sciences education because they allow exposure to potentially disturbing material in a less threatening way [36].

It should be noted that although these visual texts are not dense with information, they do provide enough detail to allow readers to identify the emotional state of characters, and begin to identify with characters [7, 32, 37]. This element of graphic novels can make them especially useful for educators to create content for affective and interpersonal/social goals, such as empathy, ethics, and communication [17, 26].

Perhaps unsurprisingly, given the strength of graphic novels and comics in conveying emotions and developing characters that can draw in their readers, graphic novels have been used in HPE to help learners to develop and maintain empathy and communicate with patients [25, 31, 38]. Graphic novels and comics build empathy because they excel in providing such learners with the perspective and experience of other people [16, 25, 31, 32, 39, 40]. Indeed, studies have suggested that the visual component of graphic novels has more of an impact on the development and sustainment of empathy than does the written component [38, 41].

The benefits of graphic novels can even extend beyond the realm of the school into lifelong learning; Myers et al. found that graphic novels were effective in fostering empathy in practicing clinicians, not just students, noting that “participants indicated that reading the book [a graphic novel about the experiences of a patient with Parkinson Disease] led to greater self-awareness, which was sometimes surprising to them” ([38], p. 4). Graphic medicine can thus play an important role in promoting professionalism [42] and a patient-centered approach to medicine [25].

Graphic novels have been used in the military environment to motivate a younger audience to engage with material that is highly technical and/or unfamiliar to them [3, 43, 44]. Navy Medicine chose to use a graphic novel as part of an initiative to reduce combat stress-related injuries, in part because they believed it would engage the enlisted personnel who were the target audience, and because the format would encourage identification with the characters and thus help reduce the stigma of seeking help for such injuries. The first graphic novel was so well-received, with high demand as well as recognition by the technical communication community, that it led them to publish a second one [7]. Likewise, a group that developed a graphic novel to help Marines begin to think about technology and the future of warfare found that the publication was so popular that they had to do multiple print runs [43].

## Leveraging Graphic Novels for Instructor Preparation

### Background

Uniformed Services University (USU) is the military’s health professions academy, charged with preparing healthcare practitioners to care for the nation’s warfighters. Central to its curriculum is the Operation Bushmaster Military Medical Field Practicum (MFP), a four-day live simulation during which approximately 200 fourth year medical students and third-year graduate nursing students apply their medical/nursing and leadership skills during a simulated deployment in an austere environment. The simulation takes place over thirteen four-hour training cycles in which the students role-play as members of a medical platoon providing support for a variety of military operations. Ten students in each platoon are in graded positions during each cycle, and students rotate through the graded and ungraded positions throughout the MFP.

The heart of the learning experience for this capstone event is feedback provided by instructors. The main instructors, each of whom is assessing two students each cycle, must provide frequent formative feedback as the students work through the scenario. At the end of each grading cycle, each instructor must also provide summative feedback to their students via a specialized assessment instrument developed by USU for MFPs, and they must also go over the assessment instrument with the students in a live debriefing session.

While skilled instructors are crucial to the success of the MFP, Operation Bushmaster presents a number of challenges for instructor preparation. Instructors possess disparate levels of clinical, teaching, and deployment experience; furthermore, many are unfamiliar with the unique assessment instrument used for this MFP. To bridge this gap, USU designed and developed a set of graphic novels to standardize and enhance instructor preparation.

USU trains the instructors by providing access to online materials in a learning management system approximately six weeks prior to the MFP, followed by a six-hour in-person orientation/training the day before the MFP begins. A structured activity in the learning management system allows the instructors to use graphic novels to familiarize themselves with the instructor role and the assessment instrument at their own pace before the orientation.

**Fig. 1:**
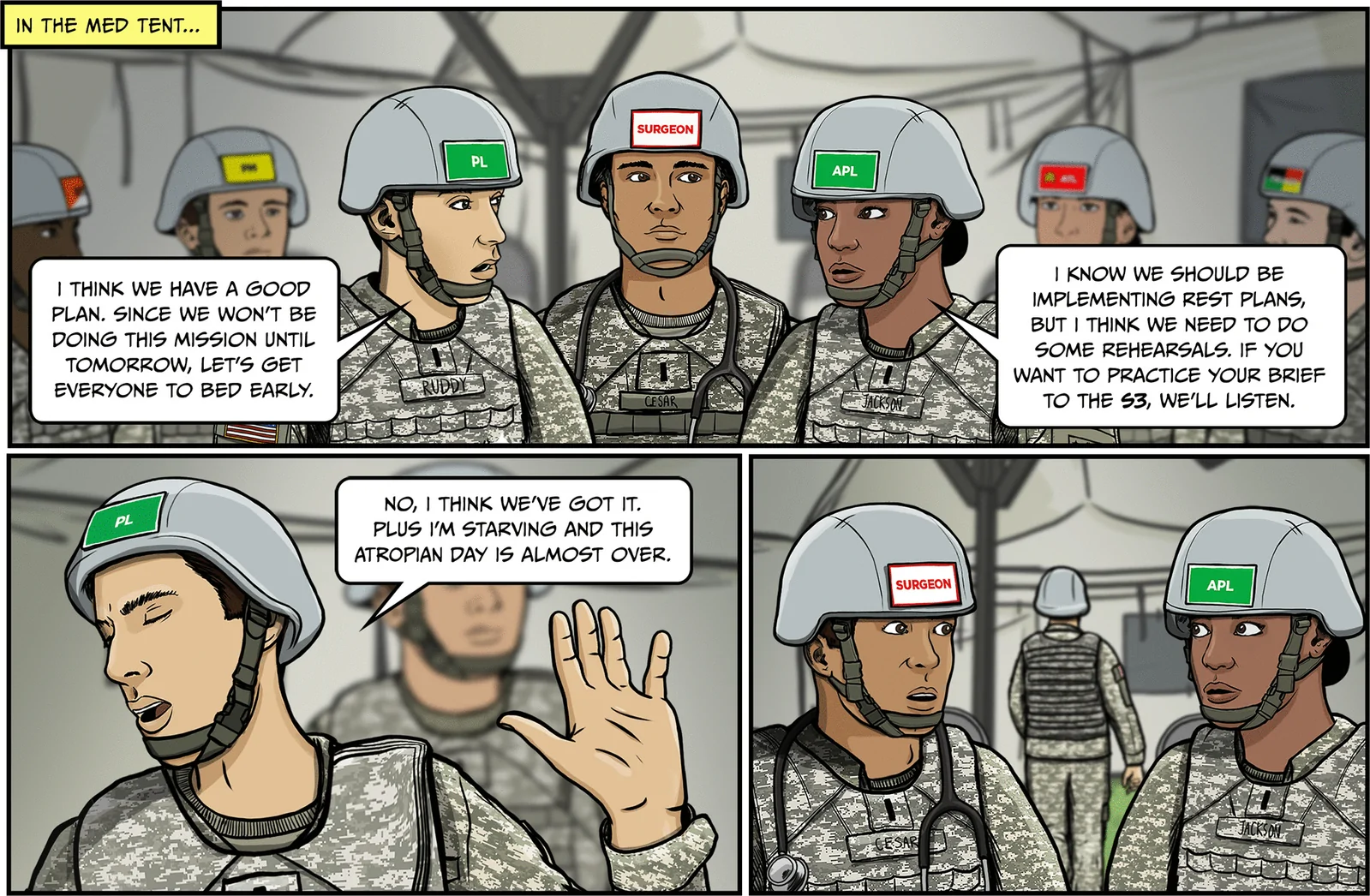
Graphic novel panels showing student in a leadership position (person with helmet labeled “PL”) interacting with peers

**Fig. 2:**
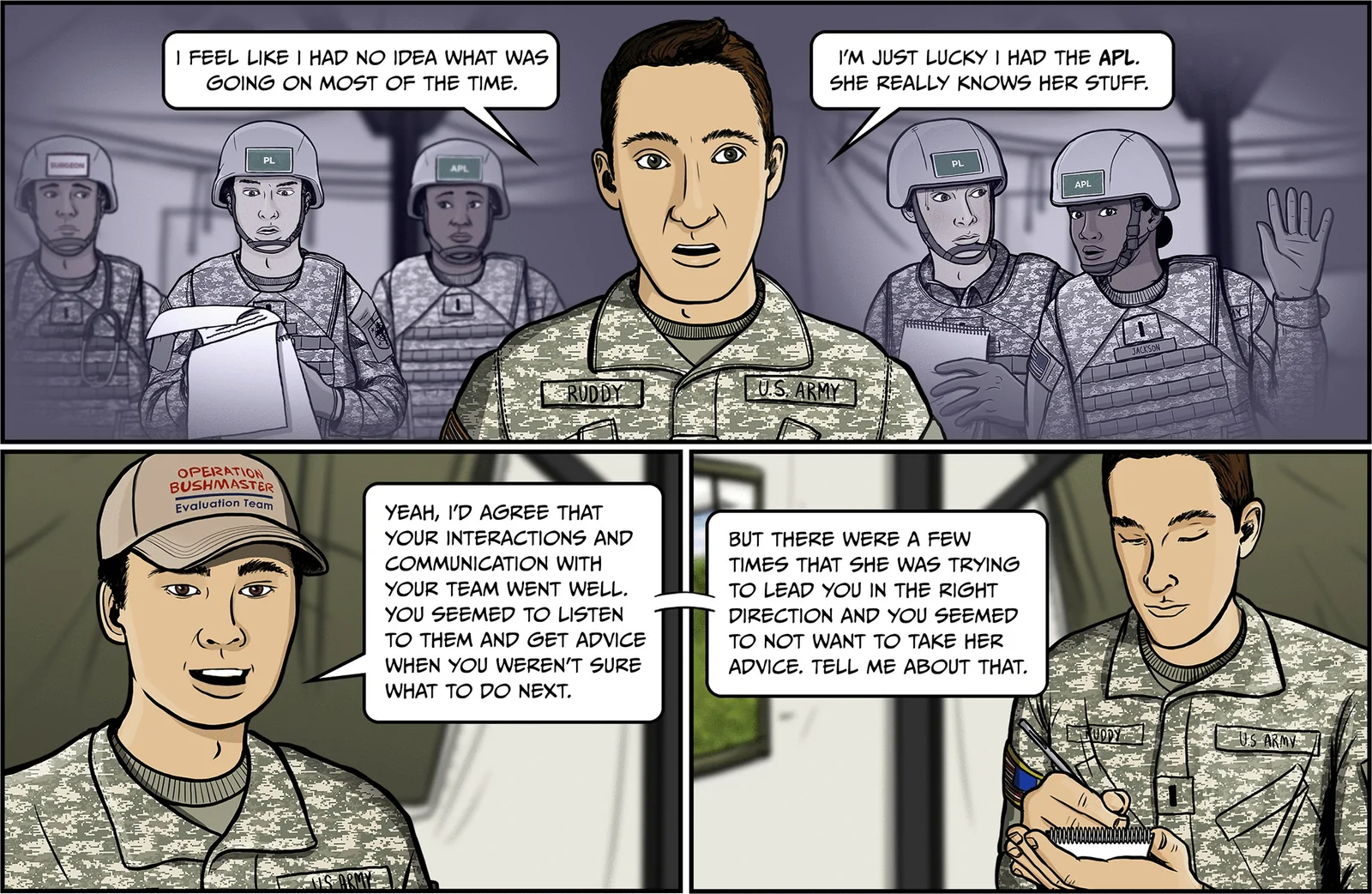
Graphic novel panels showing an instructor (wearing a hat) providing feedback to a student

The activity incorporates two different visual narratives with distinct purposes:

- **Observational Scaffolding:** Instructors review a graphic novel depicting a student in a leadership role interacting with students in their platoon. Those instructors must interpret characters’ facial expressions, gestures, actions, and positioning to complete an assessment of the student leader in a process that requires them to draw conclusions from ambiguous observations, like medical diagnosis does [19]. The graphic novel here requires readers to “fill in the blanks” and participate actively in interpreting meaning, in keeping with Short and Reeves’s [4] application of Marshall McLuhan’s theory of cool media. Figure 1, from the graphic novel for instructors assessing students in leadership positions, depicts the Platoon Leader (PL) and Assistant Platoon Leader (APL) discussing plans with the Surgeon. This panel demonstrates how the graphic novel teaches instructors about the learning environment, using relevant language (for example, “Atropian day” references Atropia, the name of the imaginary country in which the simulation takes place) and visual cues (for example, during the MFP, students wear colored patches on their helmets, as shown here, to help their instructors and their peers identify their roles) The panel goes beyond merely replicating the environment, however, to use visual cues to show the emotional complexity of the situation, providing information in a visual format which stimulates in a different manner than a written description [23, 38]. The additional information presented in the nonverbal communication depicted in Fig. 1—the PL dismissing the APL by turning away and waving his hand, and the APL and Surgeon looking at each other with surprise—requires the reader to examine the situation closely, identify the problem, and consider how it could have been handled more appropriately. Such visual complexity enables the graphic novel format to show words and actions, and the impact they have on people, from a variety of perspectives, making the graphic novel a useful tool for depicting ethical dilemmas and complex clinical scenarios in ways that challenge learners’ higher order and clinical decision-making skills.
- **Modeling of Performance:** After completing their own assessment of the student, instructors review a second graphic novel showing an experienced instructor providing feedback to that student. This provides a direct model of effective feedback techniques in action. Figure 2 illustrates a feedback session, where the instructor, in a tan hat, both provides feedback and encourages the student to reflect on his performance, as shown by the grayscale “flashback” and the instructor’s words. The distinctive visual presentation of a flashback here demonstrates the way that graphic novels can present multiple layers of reality—showing both the present and another time frame—to enable consideration of what led the character to this point, or an alternative possible future. This elegant and efficient depiction of possible alternative scenarios can be useful in depicting a character considering possible outcomes, as in a clinical scenario or an ethical dilemma.

### Perspectives on the Use of Graphic Novels for Instructor Preparation

The graphic novels, when used as a preparatory educational tool [23], provided the visual scaffolding that allowed the instructors to familiarize themselves with their role, environment and the assessment card at their own pace, fostering deeper engagement and enhancing existing capabilities.

To analyze instructor perspectives on graphic novels and identify the method’s strengths and areas for improvement, the authors collected instructor feedback via mid-MFP interviews and post-orientation and post-MFP surveys. Instructor interviews and surveys were de-identified and interviews were transcribed and responses categorized. The Institutional Review Board (IRB) determined that this protocol did not meet the criteria for research (project DBS.2025.998, ref # 987,790). Qualitative thematic analysis of the content of the categorized feedback revealed that the graphic novels helped the instructors in three main ways that align with educational uses of graphic novels described earlier in this paper: they provided environmental scaffolding, they modeled performance of the instructor role, and they allowed the instructors to calibrate their assessment. A sample of relevant comments from instructors is provided below.

- Sense of setting: The realistic drawings provided the instructors with information about the environment in which the MFP took place, enabling them to start to envision themselves in that environment.
  - One instructor commented that the graphic novels “helped to visualize … the sim[ulation] ahead of time.”
- Modeling of performance: The graphic novels’ depiction of a feedback session let the instructors see what would be expected of them when working with students and showed them good techniques to use.
  - One instructor noted that the graphic novels provided a “Good example of how to give feedback to a student with a poor performance and allowing them to discover what they did well and how to improve.”
  - Another indicated that they “Reinforced ways to provide effective feedback.”
- Calibration of assessment: Being able to compare their own assessments with those of a model instructor in the graphic novel, and see that instructor’s comments, helped the faculty to modulate their performance in the instructor role.
  - One instructor praised the graphic novels for “Setting standards—I think they made me a more reasonable evaluator.”
  - Another shared that they “Used the grading as a guide.”

The comments of the instructors after completing the graphic novel-based learning activity reinforce the strengths of graphic novels as educational materials, in keeping with CTML, communication theory, and the instructional use of scaffolding.

## From Blueprint to Production: A Methodological Framework for AI-Assisted Graphic Narrative

The creation of graphic novels for educational purposes is a complex endeavor that brings together a multidisciplinary team of graphic artists, visual design specialists, instructional designers, and subject matter experts; many of the graphic novels used for Operation Bushmaster were created in 2021 by just such a multifunctional instructional support team. However, GenAI, when used carefully, can serve as an accelerator and a force multiplier, bringing the creation of graphic novels within the reach of smaller teams, and enhancing the work of larger teams. The framework for graphic novel creation presented here identifies ways to take advantage of the strengths of both the creative personnel and the supporting GenAI tools to present complex material in a highly structured but engaging way.

This framework requires a seamless transition between two distinct stages: the Design Process and the Production Process. Architecture provides a useful metaphor for understanding the processes and the relationship between them: the Design Process encompasses the creation of a visual and narrative blueprint, while the Production Process is when construction occurs.

In order to ensure that the pedagogical intent remains intact throughout the transition from concept to final product, and to provide Quality Assurance (QA) that can mitigate the risks of misleading, inaccurate, or wholly hallucinated, GenAI content, it is crucial that the design and development process incorporates a “Human-AI-Human” (H-AI-H) approach. For this approach, the “Human” element includes both Creators, who interact with multidisciplinary team members and GenAI tools to develop different elements of the product, and a knowledgeable expert, identified in this paper as the Architect, who plans the process, reviews all material developed using GenAI, and assembles the final product. Successful development of such a product relies on the Architect leading a phased iterative approach that permits continuous testing and refinement. Rather than treating production as a linear path, a phased and stepwise model facilitates the gathering of stakeholder feedback at critical milestones, allowing for real-time adjustments that prevent minor technical issues from compounding into significant structural flaws [45].

Beyond internal logistics, Design Process and Production Process considerations should also encompass cultural and cognitive accessibility. Creators should evaluate visual assets through the lens of Universal Design for Learning (UDL) to ensure that simplified visual metaphors—while powerful for identification [46]—remain inclusive and do not create unintentional barriers for diverse student populations [47]. Finally, a forward-looking development strategy addresses visual literacy scaffolding, incorporating clear panel borders and intuitive eye-flow patterns to support learners unfamiliar with sequential art. By addressing these structural and ethical considerations, the development team ensures that the graphic novel serves as a pedagogically sound foundation for future instructional work [48].

### Phase I: The Architecture (Design Process)

Before any technical construction begins, the project must be defined through a series of conceptual documents and low-fidelity visualizations.

- Treatments: Acting as a long form written pitch, the treatment outlines the story concept and arc, establishing a compelling story with clear character objectives and educational objectives [49]. Much like in film or television, this essential document ensures the narrative foundation is sound before resources are committed to production. Consulting with subject matter experts (SMEs) during this phase allows the Design team to identify appropriate instructional content, focusing on areas where students tend to have trouble mastering the material and where it is best suited to the strengths of the graphic novel. For example, in order to use a graphic novel to present a realistic clinical encounter that would help a medical student develop their clinical reasoning skills, the Design team could consult a SME to ensure that the graphic novel presents the patient’s history and appearance in a way that would make it possible to make the correct diagnosis but that also contains enough irrelevant or ambiguous information that it makes misdiagnosis equally possible.
- Thumbnails and Sketches: These preliminary “doodles” are the quickest way to externalize ideas. Intentionally rough and unfinished, thumbnails allow the creator to map out composition and “eye-flow” without the pressure of technical perfection.
- Mood Boards: To establish the artistic direction, mood boards provide stakeholders with a sense of the aesthetic of the product. Mood boards present color palettes, typography, and reference photos for real-world accuracy. These are not final images but rather a “visual vocabulary” for the project.
- Draft Storyboards: The final step of the architecture phase is the draft storyboard, which serves as the start of the visual blueprint. At this stage the storyboard presents the initial concepts and ideas for the graphic novel.

### Phase II: The Construction (The GenAI Production Workflow)

Once the team has agreed upon the architecture and any stakeholders have approved it, the project enters Production. A structured four-step process brings the blueprint into reality. In traditional workflows, the Production Process often represents a significant hurdle. While the Design Process is driven by human creativity, the Production Process—the actual “construction” of the art—requires a level of technical labor and resource investment that can be prohibitive [7]. However, it is no longer necessary to build solely by hand; instead, high-powered GenAI tools can be used to streamline the “heavy lifting” of construction. Throughout the Production Process, however, the Architect must remain engaged to navigate the non-linear complexities of AI generation—where a single prompt adjustment can radically alter the output—to ensure all elements are working in alignment.

#### Step 1: Narrative Codification: Story and Script Development

The transition from Design to Production begins by the process of codification, the specific ways writers and artists translate an abstract idea into the unique "language" of the graphic novel. During this step, the treatment evolves from a technical document into a story that will engage readers while also educating them. What evolves is a script that takes the abstract concepts and presents them in a relatable way, using language suitable for the characters in the scenario. Large Language Models (with human oversight) excel at this type of work.

- Methodology: Using the treatments as a foundation, creators can work with GenAI to flesh out the basic storyline, prompting it with a starting point and an end point as well as any actions or events that need to take place during the story. They can also generate scripts for the characters’ conversations by providing GenAI with prompts that convey key elements of the characters’ personalities, the context of the conversation and any points that need to be made during the discussion. The more specific details that can be provided in the prompt, the more refined the output will be. See Supplement 1 for some sample prompts that could be used to generate the outline for a graphic novel.
- Script Refinement: The creators review the GenAI output and provide follow-up prompts to refine or expand it to meet the needs of the graphic novel. Since AI-generated content may be limited by the data on which it was trained, the creators may need to massage the script to make it align with the focus of the project.

#### Step 2: Visual Synthesis: Rapid Ideation and Consistency

To ensure the “Construction” phase of a graphic novel is both efficient and scalable, the development team must incorporate a consideration of long-term sustainability into its processes. To that end, the development phase prioritizes the creation of a robust Asset Library. By curating a repository of reusable character models, backgrounds, and thematic elements, teams can maintain stylistic continuity while significantly reducing future resource expenditures. This is particularly vital in specialized fields like graphic medicine, where leveraging existing visual assets ensures a consistent aesthetic across diverse educational products.

During this step, creators first break the story down into a technical layout, dividing content into pages, panels, and dialogue for each. Careful work during story and script development ensures that dialogue and panel breaks are optimized for cognitive processing, preventing the duplication of visual and textual information. Gen AI can then be used to accelerate the generation of artwork, which is the most time-intensive part of graphic novel creation. The goal is to move from a script to a visual narrative through a controlled, professional workflow with QA provided by the team’s Creators rather than allowing the GenAI to randomly generate the artwork.

- Methodology: Creators work with AI to establish character references, generating high-quality images of characters in neutral poses to serve as anchors. Many advanced GenAI tools allow these to be used as “Character References” to maintain a unified look across all panels. See Supplement 1 for some sample prompts that could be used to generate styleframes and images for a graphic novel.
- Style Standardization: In this stage, the creator works with the GenAI to flesh out detailed textual descriptions of character appearances, outfits, and key locations. These descriptions act as the “source code” for visual consistency in the steps to follow. By including a specific style prompt (e.g., “American comic book style” or “ink illustration”) in every image generation, the creator ensures the “bricks” of the house all match the initial mood board.

The output of this stage is the final layout. It adds to the draft storyboard the refinements provided in this stage, and it also provides details about sequences, viewpoints, and transitions, allowing the Architect to anticipate potential production problems before they arise, and it aligns artwork with specific educational goals. While the AI construction workflow significantly accelerates asset generation, the Architect must remain vigilant of the stress this puts on teams, finding the “sweet spot” that allows for rapid iteration without stifling the agency of the creators.

#### Step 3: Compositional Management: Continuity Tools

During this step, creators use specialized features of graphic editing software to maintain continuity within and standardization within the elements of the graphic novel.

- Methodology: If a specific pose is required, creators can upload a simple thumbnail sketch or reference photo for the GenAI to follow, ensuring the final output adheres to the intended composition. Creators must conduct QA reviews of the work to ensure the GenAI follows the specific vision laid out in the storyboards
- Inpainting/Outpainting: These tools are used for “corrective surgery,” allowing for minor adjustments or the extension of a background without regenerating the entire panel.

#### Step 4: Final Assembly and Post-Production

The final step returns the project to human-centric design software to assemble the parts into a whole. Following multimedia learning principles, the Production Process must ensure that textual elements complement rather than duplicate visual information to maximize cognitive processing [50].

- Methodology: GenAI-produced images are arranged into page layouts. Speech bubbles, captions, and special effects (SFX) are added manually using graphic editing software to ensure the dialogue is legible and the narrative rhythm is preserved. In a static medium, SFX are visual metaphors used to represent sound, motion, and energy that the reader cannot hear or see moving.
- Refinement and Polishing: The final step involves adjusting cropping, brightness, and color grading across the entire graphic novel. This ensures a unified look, addressing the resource-intensive nature of high-quality production while ensuring learning and narrative outcomes are met [7].

By buffering for the time required to refine AI-generated content and conduct “corrective surgery” on complex panels, the Architect ensures that the final artifact is not merely a collection of high-fidelity images, but a coherent, high-quality educational tool.

## Limitations

While the examples and pedagogical frameworks detailed herein offer a promising frontier for educational use of graphic novels in the context of professional military education and HPE, several critical limitations warrant acknowledgement.

First, the study itself focused on a single academic activity and had limited measurements of effectiveness; expanding the use of graphic novels to other activities and measuring learning and performance outcomes could provide more robust evidence of the effectiveness of graphic novels.

Additionally, the efficacy of the graphic novel medium hinges on the assumption of uniform visual literacy. The cognitive demands of decoding multimodal text varies widely: for learners lacking foundational visual literacy skills, the medium may inadvertently introduce cognitive overload rather than alleviate it, potentially obscuring crucial instructional cues [38, 51, 52]. Educators planning to use graphic novels need to ensure that the visuals are carefully designed to emphasize, rather than distract, from the primary goals, and may want to provide learners with guidance on how to “read” this medium [41].

Another potential barrier to the use of graphic novels and comics in HPE is a belief that some might find them unsuitable for use in a formal educational setting [42]. However, studies have found that, once they have been exposed to graphic novels in a course, medical and nursing students enjoy them, find them useful, and would like to use similar material in the future [23, 24, 32].

Beyond these challenges inherent in the medium of the graphic novel, the integration of artificial intelligence in the creation of educational content introduces distinct limitations regarding quality assurance and ethical stewardship. Where instructional clarity directly impacts physical safety, as can be the case in HPE, the potential for bias in algorithms or kinetic inaccuracies in AI-generated visual outputs represents a formidable risk. Many organizations lack standardized QA protocols capable of vetting such media without exhaustive expert intervention or major skill in graphic production. Finally, deploying AI modalities necessitates rigorous ethical oversight, particularly concerning algorithmic transparency, copyright and intellectual property, as well as the expenditure of academic and natural resources [53, 54] As researchers we recognize that establishing boundaries on these issues via organizational policies provides essential guardrails defining the critical parameters for future empirical studies and ethical governance in research.

## Conclusion

USU’s experience preparing instructors for high-stakes simulations aligns with research that illustrates how graphic novels, with their ability to depict a range of human interactions in a specific environment can serve as an effective instructional scaffold for HPE.

By integrating sequential art with written text, graphic novels provide a unique medium for teaching complex concepts and exploring critical thinking. While graphic novels used to be the province of those who were able to devote significant resources to their development, the emergence of GenAI has brought the creation of compelling graphic novels within reach of organizations that have limited instructional and graphic design capacity. By following a structured design and production workflow—leveraging AI for narrative codification and visual synthesis—educators can efficiently build consistent, high-fidelity narratives. When managed through a “Human-AI-Human” ecosystem, these graphic novels offer a promising foundation for supporting the development of the leadership, communication, and empathy skills required in modern healthcare. 

## Supplementary Information

Below is the link to the electronic supplementary material.

Supplementary file1 (DOCX 22 KB)

## Data Availability

Not applicable.

## Declarations

### Disclaimer

The opinions and assertions expressed herein are those of the author(s) and do not reflect the official policy or position of the Uniformed Services University of the Health Sciences or the Department of War.

### Rights and Permissions

The excerpts from the graphic novel that are presented in the article are works of the Education & Technology Innovation Support office at the Uniformed Services University of the Health Sciences, a part of the US Government’s Department of War, and are thus in the public domain.
