# Supplementary material for: A Picture is Worth 1000 Words: Using Graphic Novels to Support Health Professions Education
Source: Med Sci Educ. 2026 Oct 1;36(6):2999–3008. doi: 10.1007/s40670-026-02871-9 (PMC13759677; doi:10.1007/s40670-026-02871-9)
Supplement: Supplementary file 1 [file 40670_2026_2871_MOESM1_ESM.docx]

# Supplemental Information: Sample Prompts

## Sample Prompt to Generate an Outline for a Graphic Novel

When using AI to generate an outline / script for a graphic novel, provide it with as much information as possible about the story you would like to depict. For example:

- Describe the setting and characters.
- Identify the starting point and end point for the plot, and any intermediate scenes you want to include.
- Specify how many pages you would like the graphic novel to be, and how many panels you would like to include on each page.

Below are two sample prompts that have the same general focus but slightly different points of emphasis:

**Sample Prompt 1:**

Act as a graphic novel writer. Create a detailed story outline based on the information below that results in a graphic novel that contains no more than 16 pages, each with 4 or 6 panels.

Include:

1. Starting Point: The protagonist's initial mindset and visual tone.
2. Ending Point: The protagonist's final perspective shift.
3. Scene-by-Scene Actions: For each scene, list the key events, physical actions, and crucial dialogue moments.

Characters:

- A brash, overconfident 4th-year military medical student acting as Ambulance Team Leader (ATL).
- Team: 1 seasoned Medic and 3 Litter-Bearers.

Scenes:

- Scene 1: The ATL gives a vague, high-level plan to the team, skipping key details and load-plan rehearsals.
- Scene 2: An emergency call comes in. The ATL gives loose packing guidance—forgetting pain medication and stretchers (and failing to plan how to load patients into the Field Litter Ambulance).
- Scene 3: At the Point of Injury, the team realizes equipment is missing. The ATL gets frustrated and blames the situation.
- Scene 4: Handoff at the aid station. The ATL must explain to the Surgeon why the patient had no pain meds and was hand-carried to the ambulance.
- Scene 5: Debrief with faculty. The faculty asks the ATL to self-reflect on what went well and where they need to improve.

**Sample Prompt 2:**

You are an expert Graphic Novel Scriptwriter and Visual Storyboard Director specializing in high-tension, realistic military drama and medical narrative arcs.

Your task is to take the provided story concept and transform it into a comprehensive Graphic Novel Narrative & Action Outline.

CHARACTER BRIEF

- Protagonist: 4th-Year Military Medical Student (Ambulance Team Leader / ATL).
  - Personality: Brash, overconfident, academically smart, but practically naive and dismissive of logistics and real-world friction.
- Secondary Characters:
  - 1 Experienced Medic (skeptical, knows field realities).
  - 3 Litter-Bearers (execution-focused team).
  - 1 Attending Trauma Surgeon (exacting, experienced, demanding precision).
  - 1 Faculty Evaluator (calm, insightful, reflective).

Please generate a structured, highly detailed Graphic Novel Outline containing the following four sections:

1. NARRATIVE ARC OVERVIEW
   a. Starting Point: Define the protagonist's mindset, visual demeanor, and status at the opening frame.
   b. Ending Point: Define the protagonist's shifted perspective, emotional posture, and realization at the closing frame.
   c. Core Visual Motif/Theme: Suggest a repeating visual symbol or contrast motif (e.g., equipment checklists vs. chaos, clean lights vs. mud).
2. GRANULAR SCENE-BY-SCENE ACTION & EVENT OUTLINE
   For each of the 5 scenes, provide:
   a. Setting & Mood: Lighting, atmosphere, environmental tension.
   b. Key Physical Actions & Events: Step-by-step actions that occur on page (e.g., specific gear mishandling, radio calls, physical struggles).
   c. Key Dialogue Beats: Crucial lines that drive character conflict or reveal mistakes.
   d. Visual/Panel Highlights: Specific key art moments (e.g., extreme close-ups, wide shots, split panels).
3. LOGISTICAL & DRAMATIC FAILURE POINTS
   a. Explicitly break down the cascading chain of mistakes made by the ATL (from skipped rehearsal to missing stretchers to failed handoff) so the artist can visually emphasize cause-and-effect.
4. PAGE & PACING RECOMMENDATIONS
   12-16 total pages

*******************************

Sample Prompt to Generate Graphics for a Graphic Novel

It is possible to generate a graphic novel by following up on one of the outline requests above with the following prompt:

Using that outline, generate the images that depict the action and insert the dialog to create a complete graphic novel.

However that output may look “mass-produced” or not be quite what you were expecting, and you may not be able to edit it.

If you would like to “team up” with AI so that AI creates the foundation and you have more control over the individual elements of the graphics, you might want to take a phased approach.

First, draw sketches of what you have in mind and use AI to create a style anchor and convert the sketches into clean illustrations. Be as specific as you can about the style and any image effects and colors you want.

Below is a sample prompt:

Using the attached image [uploaded sketch], convert the composition into a clean, flat 2D graphic illustration styleframe. Apply a subtle risograph grain texture across all shapes. Use only clean, sharp vector edges and simplified, geometric forms. Strictly enforce a restricted color palette of [COLOR 1], [COLOR 2], and [COLOR 3]. Do not include any realistic elements, 3D effects, gradients, shadows, and do not include textures from the original sketch lines. The final aesthetic must have a pure 2D vector essence.

Keywords to emphasize: Flat 2D illustration, minimalist vector art, screen-printed aesthetic, risograph texture, matte finish, simplified geometric shapes, infographic style.

Negations to use: Realistic, 3D render, soft gradients, pencil texture, photorealistic.

Be prepared to provide feedback and iterate until you get the style you want.

Once a good styleframe is generated, you can upload that image into the AI tool along with different scenarios based on the script and prompt it to convert the new composition into the exact style, texture, and color palette of the successful reference image.

You can use the AI output as-is, modify the images using graphic software, or create images yourself using the AI images and styleframe as a guide.

Article Information

**Article title**: A Picture is Worth 1000 Words: Using Graphic Novels to Support Health Professions Education

**Journal name**: Medical Science Educator

**Author names**: Karen (Beth) Marcellas, PhD; Melissa Myers, MD; Dina Kurzweil, PhD

**Affiliation and e-mail address of the corresponding author**:
Karen. (Beth) Marcellas, PhD

Uniformed Services University of the Health Sciences (USU)

Education & Technology Innovation (ETI) Support Office

Bethesda, MD, USA

[karen.marcellas@usuhs.edu](mailto:karen.marcellas@usuhs.edu)
